# Biosynthesis and Role of N-Linked Glycosylation in Cell Surface Structures of Archaea with a Focus on Flagella and S Layers

**DOI:** 10.1155/2010/470138

**Published:** 2010-10-05

**Authors:** Ken F. Jarrell, Gareth M. Jones, Divya B. Nair

**Affiliations:** Department of Microbiology and Immunology, Queen's University, Kingston, ON, Canada K7L 3N6

## Abstract

The genetics and biochemistry of the N-linked glycosylation system of Archaea have been investigated over the past 5 years using flagellins and S layers as reporter proteins in the model organisms, *Methanococcus voltae, Methanococcus maripaludis,* and *Haloferax volcanii*. Structures of archaeal N-linked glycans have indicated a variety of linking sugars as well as unique sugar components. In *M. voltae, M. maripaludis,* and *H. volcanii*, a number of archaeal glycosylation genes (*agl*) have been identified by deletion and complementation studies. These include many of the glycosyltransferases and the oligosaccharyltransferase needed to assemble the glycans as well as some of the genes encoding enzymes required for the biosynthesis of the sugars themselves. The N-linked glycosylation system is not essential for any of *M. voltae, M. maripaludis,* or *H. volcanii*, as demonstrated by the successful isolation of mutants carrying deletions in the oligosaccharyltransferase gene *aglB* (a homologue of the eukaryotic Stt3 subunit of the oligosaccharyltransferase complex). However, mutations that affect the glycan structure have serious effects on both flagellation and S layer function.

## 1. Introduction

N-linked glycosylation is one of the most common posttranslational modifications found on proteins in eukaryotic cells [1] and has now been documented in both prokaryotic domains as well [2, 3]. Searches of complete genome sequences can readily identify homologues to the oligosaccharyltransferase STT3 subunit that transfers the assembled glycan from a lipid carrier to the target motif (amide linkage to asparagine within the sequon N-X-S/T) on the protein. This gene would be required in all organisms where N-linked glycosylation occurs and is readily found in eukarya and a limited number of bacteria but in almost all sequenced archaeal genomes. Of greater than 50 completed archaeal genomes, only 2 appear to lack this gene (*Aeropyrum pernix* and *Methanopyrus kandleri*) [4], suggesting that this posttranslational modification is much more common in archaea than in bacteria. Indeed, while many glycans associated with S layers in Bacteria have been reported, all are exclusively O-linked [5]. In Archaea, where glycosylation of S layers is more common than in Bacteria, most glycan linkages are of the N variety [6], although S layers containing glycans attached by both O and N linkage occur [7]. Extremely little is known of the O-linked process in Archaea. 

In Archaea, N-linked glycosylation is most commonly found on S layer proteins [3, 6, 8–12] and flagellins [7, 9, 10, 13, 14] and, more recently, on another surface protein, pilin (unpublished data). However, there are other proteins that have been shown in a variety of archaeal species to be N-glycosylated that are unrelated to those commonly N-glycosylated proteins. These include a hexasaccharide-modified cytochrome b_558-566_ in *Sulfolobus acidocaldarius* [15] and a cytoplasmic membrane protein in *Thermoplasma acidophilum* containing a highly branched glycan, composed mainly of mannose that is N-linked through an N-acetylglucosamine [16]. In addition, it was shown in *Pyrococcus furiosus* that purified oligosaccharyltransferase could transfer a lipid-linked heptasaccharide prepared from *P. furiosus* cells to an Asn within a sequon Asn-X-Thr/Ser contained in a peptide substrate [17]. The role that the N-linked glycan plays is uncertain but what is known is that underglycosylation or nonglycosylation of these proteins can have significant effects (see below), even though for *Methanococcus voltae, Methanococcus maripaludis,* and *Haloferax volcanii,* knockouts of the oligosaccharyltransferase have been reported [18–20] indicating that the N-linked process is not an essential one for either of these model archaea. 

In this contribution, N-linked glycosylation structures, assembly, biosynthesis, and role in archaeal surface structures are reviewed.

## 2. Surface Appendages in Archaea

Similar to bacteria, the existence of surface appendages on archaeal cells has been known for a long time [21–23]. Some structures, like archaeal flagella and pili, show similarities to their bacterial counterparts in appearance, while several other structures like cannulae, hami, the newly discovered *Ignicoccus* fibers [24], and the putative bindosome appear to be novel structures found, thus far, only on archaeal cells [23]. 

### 2.1. Flagella

Archaeal flagella are rotating organelles with a filament and hook as seen in bacteria, but they do not show any similarity to the bacterial flagella in terms of their component parts or assembly [25–29]. The flagella of archaea are often in the 10–12 nm diameter range, much thinner than typical bacterial flagellar diameters. The flagellin structural proteins are typically 200–240 amino acids long, although there are some significantly longer. Archaeal flagella, the most thoroughly studied of the archaeal appendages, are only swimming, but also involved in cell-cell interactions and in the initial attachment to surfaces as a prerequisite for biofilm initiation in certain archaea [30, 31]. 

Flagella have been reported in all of the major subgroupings of cultivatable archaea, such as halophiles, haloalkaliphiles, methanogens, hyperthermophiles, and thermoacidophiles [27, 32]. Detailed studies have been reported in a variety of archaeal genera, including *Methanococcus *[33, 34], *Halobacterium* [35–37], *Sulfolobus* [38], *Natrialba* [39], *Thermococcus *[40], and *Pyrococcus* [30]. Even though they are superficially similar to bacterial flagella in appearance, the archaeal flagellum is a unique motility apparatus that has a well documented similarity to bacterial type IV pili [32, 41–44]. These similarities include structural ones as well as the presence of a number of genes that are conserved between the two systems. Early observations indicated a sequence similarity of archaeal flagellins and type IV pilins at their N-termini [45] and the presence of type IV pilin-like signal peptides on archaeal flagellins [33, 46, 47]. Later studies revealed conserved proteins in both systems, including an ATPase [48], a conserved membrane protein [49], and a signal peptidase (FlaK/PibD) [46, 47, 50, 51]. 

Flagellated archaea generally possess a single major identified genetic locus which encodes the flagellins and a number of conserved *fla* genes, including *flaH*, *flaI,* and *flaJ* that are found in all flagellated archaea. Between the flagellin genes and *flaHIJ* can be a variable number of other genes from among *flaC, D, E, F,* and *G*. Genetic studies have shown that these genes, as well as the conserved *flaHIJ*, are essential for flagella assembly even though not all flagellated archaea possess them [52–55]. FlaI, a homologue of the extension and retraction ATPases in the type IV pili systems, has been shown to display ATPase activity [56] and exist as a hexamer [57]. FlaH is another possible ATPase: it contains a Walker Box A but not a Walker Box B [58] but no studies on its possible ATPase activity have been reported. FlaJ is similar to the conserved type IV pilus system membrane component PilC/TadB and may form part of the platform for assembly of the flagella [49, 59]. An additional genetic similarity to the type IV pili system is the presence of a prepilin peptidase homologue gene, the preflagellin peptidase *flaK/pibD*, usually located elsewhere on the chromosome from the main *fla* operon, which encodes an essential signal peptidase required for flagellin processing. It is a member of the same novel aspartic acid protease family of enzymes as the prepilin peptidase. 

While numerous similarities of archaeal flagella to type IV pili have been presented, other fundamental differences that clearly differentiate archaeal from bacterial flagella have been identified. Recently, it was shown that the rotation of archaeal flagella is powered by ATP hydrolysis and not by the proton motive or sodium motive force used by bacterial flagella [60]. In addition, a key structural feature that makes the archaeal flagellum unique is the lack of a central channel [43, 44]. Hence, it was clear that the assembly of the archaeal flagella could not take place by addition of flagellin subunits traveling from the base through the hollow structure to final incorporation at the distal tip, as seen in the well-studied type III secretion system used for assembly of bacterial flagella [61] but most likely occurred by subunits added at the base [26, 32]. See below *(model of assembly of archaeal glycosylated flagellins into flagellar filaments)* for more on assembly of archaeal flagella. 

Glycosylation of archaeal flagellins appears to be a widespread posttranslational modification [62]. Unlike the case of bacterial flagellins where there are occasional reports of flagellin glycoproteins but always with an O-linked glycan [63], archaeal flagellins are modified with an N-linked glycan. A three- or four-sugar glycan has been reported on *Methanococcus* flagellins [9, 13, 64] and this glycan attachment has profound effects on the assembly and functioning of the flagella (see below).

### 2.2. Pili

Pili are among the most common surface appendages found on archaeal species and have been reported for decades [21, 22]. However, there is still only limited information available on these appendages although structural, functional, and genetic studies have recently been published on pili from several archaeal species. At least two different types of pili structures have been reported in Archaea: ones that are assembled from type IV pilin-like proteins, as seen in several genera including *Methanococcus* and *Sulfolobus,* and pili that are not seemingly related to type IV pili, as in *Methanothermobacter thermoautotrophicus*. A study on *M. thermoautotrophicus *fimbriae/pili indicated that the main structural component was a 16 kDa glycoprotein (Mth60) with no known homologues reported in the databases [65]. For the first time in Archaea, pili structures were shown to be adhesive surface appendages, and their abundance on the cell surface was greatly enhanced when cells were grown on surfaces rather than in liquid cultures [65]. 

 Recent studies on *S. solfataricus* have reported that a type IV pilus-like operon, including an ATPase, membrane component, and two pilin-like genes was strongly upregulated upon UV light irradiation [66, 67]. Pili-like structures termed Ups pili, 10 nm in diameter and peritrichously located on the cell surface, were found following UV treatment, and these pili were necessary for a subsequent cellular aggregation which may enhance DNA transfer, helping cells recover from UV induced DNA damage [67]. Deletion of the ATPase found in the pilin operon leads to nonpiliated cells, confirming that this locus was responsible for the observed Ups pili [67]. Ups pili were also shown to be essential, along with flagella, for initial attachment to surfaces, and upregulation of genes involved in pili formation was demonstrated upon attachment of cells [31]. 

Pili on the surface of *M. maripaludis* are much less numerous than flagella. Pili from *M. maripaludis* are composed of a major structural protein of 17 kDa. Using cryo-electron microscopy, it was shown that these pili have a structure unlike that of any known bacterial pili, with two subunit packing arrangements coexisting within the same pilus [68]. A genetic locus, predicted to be involved in type IV pili-like structures, was identified [69], and subsequent genetic knockout work has confirmed their involvement with pili formation, although the apparent major structural pilin is encoded outside this locus and is only 62-amino acid long after signal peptide removal (unpublished results). This locus is also unusual in that the assembly ATPase is not located there but is instead located elsewhere on the chromosome (unpublished results). The locus contains 3 pilin-like genes and an associated prepilin-like peptidase, EppA. All prepilins are potentially glycoproteins, with the major structural pilin proven to have an N-linked glycan similar to the one found on flagellins but unexpectedly with an additional, branched sugar (unpublished results). It was shown in coexpression studies that EppA was able to process the prepilins but not the preflagellins while the preflagellin peptidase FlaK was able to process preflagellins but not the prepilins, even though both precursors have type IV prepilin-like signal peptides and the amino acids surrounding the cleavage site are quite similar [69]. Interestingly, in *Sulfolobus* a single prepilin peptidase-like enzyme, PibD, processes the flagellins, pilins, and sugar-binding proteins and has been demonstrated to have a less restrictive requirement for its substrates than FlaK [50, 70].

As more archaeal species are examined in detail, it seems inevitable that additional fundamentally different types of pili will be discovered, as has been demonstrated already in bacteria.

### 2.3. *Cannulae, Hami, Iho670 Fibers*, and the *Bindosome*

Other surface structures have been reported in Archaea, although they are currently not as well studied as flagella and pili, often because they are found in species without tractable genetic systems. One such surface structure is a network of tubules, termed cannulae, which have only been found so far in the genus *Pyrodictium*. Cannulae are hollow tubes that are highly resistant to heat and denaturing agents [71]. As with many archaeal surface structures, they are composed of glycoproteins, in this case three homologous glycoprotein subunits, although the nature of the glycan linkage has not been reported. Evidence has shown that cannulae act as an intercellular connector of periplasmic spaces between different cells [72]. Although the precise function of cannulae is unknown, it is assumed that they anchor cells to each other and they may be involved in the exchange of nutrients or even genetic material between cells. 

Another appendage, recently reported in a formally undescribed archaeon isolated from a cold sulfidic spring, is the novel filamentous structure termed the hamus. Hami are appendages of remarkably high complexity with the appearance of barbed wire ending in a grappling hook 60 nm in diameter [73]. Each cell is surrounded by about 100 hami. Hami are stable over a wide temperature range (0 to 70°C) and pH range (0.5 to 11.5) and are composed of subunits of 120 kDa that did not stain with the glycoprotein-staining periodic acid Schiff (PAS) reagent. They mediate strong cellular adhesion to surfaces of different chemical compositions as well as adhesion between cells themselves. Hami were also shown to be the major protein component of an archaeal biofilm, where cells form a regular three-dimensional arrangement kept a constant distance apart from each other, likely through the activity of the hami [74]. 

The bindosome is a putative archaeal structure with a unique function in *S. solfataricus* [75]. The main structural components of the bindosome are substrate (sugar) binding proteins made with type IV pilin-like signal peptides that are cleaved by the same prepilin peptidase homologue (PibD) that removes the signal peptides from both flagellins and pilins in this organism. Hence, the bindosome has been proposed to be a pilus-like structure located near the surface of the cell [76]. Consistent with many other archaeal surface structures, the substrate binding proteins are known to be glycoproteins [77]. Among the sugars identified in the glycan was N-acetylglucosamine, often found as the sugar linking a glycan to asparagine in N-linkages. The sugar binding proteins do contain potential sites for N-glycan attachment, although the actual linkage of the glycan has yet to be reported. FlaI and FlaJ paralogs, BasE and BasF, appear to be involved in the biogenesis of the bindosome structure. Additionally, three small genes *basA*, *basB,* and *basC* encoding proteins with type IV pilin-like signal peptides have also been identified. Deletion of these genes results in slow growth in media containing glucose and arabinose suggesting that these proteins have an accessory role in the assembly of the bindosome, perhaps sharing a function with the minor pilins in type IV pili assembly [78]. 

 Very recently, a new cell surface appendage named the “Iho670 fiber” was detected on the surface of *Ignicoccus hospitalis* [24]. The Iho670 fibers are extremely brittle structures that are distinct from flagella and pili in the primary structure of the constituting protein and the fact that they are not motility organelles. Interestingly, the major component protein is synthesized with a type IV pilin-like signal peptide and processed by a prepilin peptidase homologue. While the isolated protein did not stain positively as a glycoprotein using the PAS stain, it has been previously observed that a negative PAS staining reaction also occurred with some archaeal flagellins that were later shown to be glycoproteins [9, 13, 79]. The fiber protein has potential N-linked sequons, and *I. hospitalis *does have an STT3 (oligosaccharyltransferase) homologue (Igni_0016; [4]). Since a type IV pilin-like system is also used for flagella, certain pili, and sugar binding proteins (the bindosome) in archaea, it may be a very widespread pathway used by archaea for assembling surface structures.

### 2.4. S Layers

S layers are a very common component of cell envelopes of prokaryotes in general but are especially prevalent on the cell surface of archaeal species [80, 81]. S layers are monomolecular crystalline arrays of protein or glycoprotein subunits. While not appendages, they are, nonetheless, important extracellular components that are routinely composed, in archaea, of glycosylated proteins [5, 82, 83]. In many archaea, S layers are the sole component of the cell envelope since archaea lack the typical bacterial wall component, murein. In these archaea, the S layer interacts directly with the underlying cytoplasmic membrane in an envelope structure unseen in bacteria [84]. Many archaeal S layers have been shown to be composed of glycoproteins, with many having N-linked glycans or a mixture of N- and O-linked glycans [5, 7, 85]. This is in contrast to bacterial S layer glycoproteins which have only O-linked glycans [1, 5]. Many different functions have been postulated for S layers, including acting as a molecular sieve and, in archaea, as a shape maintaining structure [84, 86, 87]. In archaea, S layers often have long hydrophobic protrusions that integrate into the cytoplasmic membrane and result in a kind of periplasmic space between the canopy of the S layer and the underlying cytoplasmic membrane [88].

## 3. Comparisons of N-Glycosylation in the Three Domains

Before delving into the specific N-linked glycans identified in Archaea and their assembly and role in archaeal surface structures, it is important to consider the similarities and variations in the N-linked glycosylation systems in the three domains of life, Eucarya, Bacteria, and Archaea (Table 1).

The very first prokaryotic protein shown to be a glycoprotein was the S layer (cell surface glycoprotein, CSG) of *Halobacterium salinarum *[3], a historically important discovery that dispelled the longstanding belief that only eukaryotic cells had the capability to glycosylate proteins. Now, a well-defined model system for the bacterial process has been reported in *Campylobacter jejuni* [2, 89]. The fact that the *Campylobacter pgl* system can be functionally transferred to *E. coli* has greatly aided its dissection [89]. Comparisons of the bacterial system to that of eukaryotic cells show obvious similarities as well as significant differences between these two domains with the Archaea adding even more variation. 

Thus far, it seems that very few bacteria outside of the Campylobacterales appear to have an N-glycosylation system and only in *Campylobacter* species has the system been dissected. Among the bacteria, strong BLAST hits to the oligosaccharyltransferase PglB (complete with the conserved catalytic WWDXG motif) can be found in the genomes of *Wolinella *and *Desulfovibrio* [90] as well as *Sulfurovum* sp. NBC37-1, *Nautilia profundicola, Sulfurospirillum deleyianum, Sulfurimonas denitrificans, Caminibacter mediatlanticus, Nitratiruptor sp. SB155-2, *and *Helicobacter pullorum*, among others. It appears that the N-linked glycosylation system is not as restricted as originally thought, and there appears to be little doubt that demonstration of N-linked glycosylation systems in other bacteria will soon be reported. In contrast, the N-glycosylation process appears to be almost universal in archaea where a readily identified oligosaccharyltransferase (*aglB*) required for the transfer of the completed glycan from a lipid carrier to the target protein [18] is found in almost all sequenced genomes [4, 91]. Interestingly, recent analysis of the comparative structure of oligosaccharyltransferases representing all three domains shows three types of catalytic centers. While a description of the differences in the three catalytic centers lies outside the boundaries of this paper, the interesting observation is that all eukaryotic oligosaccharyltransferases have a single type (E-type) and the bacterial PglB has a single different type (B-type) while different archaeal AglB divide into three groups (E, B, and A-type (archaeal type)), each with a different catalytic center and thus showing much more capacity for variability than the other two domains [91]. 

The N-glycosylation systems of Eucarya, Bacteria, and Archaea all are thematically similar, although all have distinctive traits (Table 1). In all organisms, the glycan is assembled sugar by sugar by specific glycosyltransferases using nucleotide activated sugars as substrates. The lipid carrier is undecaprenol diphosphate in Bacteria [92] while it is a dolichol derivative for both Eucarya and Archaea. In Eukarya, the carrier is dolichol pyrophosphate while in Archaea both the pyro and a monophosphate dolichol have been reported [93–95]. Early work in *Halobacterium salinarum* where there are, unusually, two different N-linked glycans attached to the S layer, indicated that the repeating unit glycan was assembled on a dolichol pyrophosphate carrier while the oligosaccharide glycan was assembled on a dolichol monophosphate carrier [96].

Assembly of the glycan occurs on the cytoplasmic face of the cytoplasmic membrane for *Campylobacter,* and the completed glycan is flipped by an ATP-dependent flippase, PglK [97], to the periplasmic side prior to its transfer to the target protein. In eukaryotic cells, the glycan is assembled on the cytosolic side of the ER membrane before being transferred to the lumen side where further additional sugars are added from dolichol-P sugars. The transfer to the lumen of the ER was assigned to the ATP-independent flippase Rft1 [98] although recent evidence casts doubt on that original assignment [99]. Recent work has indicated that the topology of the N-glycosylation system in archaea is also with the assembly of the glycan on the cytoplasmic side of the cytoplasmic membrane [100] although the putative flippase remains unidentified. No Rft1 homologues appear in the genomes of *M. maripaludis* while BLAST searches using PglK retrieve many strong hits due to the presence of many ABC transporters. Targeted deletions of likely candidates have so far failed to reveal the elusive flippase in archaea [18, 19]. 

The linking sugar connecting the glycan to the target Asn in the Asn-X-Thr/Ser motif is also different in the three domains. In the bacterial (*Campylobacter)* system, this is di-N-acetyl bacillosamine [101] (an early report of a Asn-rhamnose linkage for a glycan N-linked to the S layer of *Bacillus stearothermophilus* [102] was later corrected [103]) while in eukaryotic cells this is typically N-acetylglucosamine, although glucose is used in laminin [104]. In archaea, several different linking sugars have been reported in the limited structures so far determined, both N-acetylglucosamine and N-acetylgalactosamine as well as simple hexoses [9, 13, 105, 106]. The transfer of the glycan to the target protein is carried out by an oligosaccharyltransferase. In *Campylobacter,* this is PglB, and when the system is transferred to *E. coli* [89], PglB can transfer a variety of unnatural glycans to the *C. jejuni *acceptor protein AcrA but all the identified substrates contain either bacillosamine (the natural substrate), N-acetylglucosamine, N-acetylgalactosamine, or N-acetylfucosamine at the reducing end, that is, they all contain an acetamido group at position 2 [107]. A similar conclusion was reached for the eukaryotic oligosaccharyltransferase [108], although this seems at odds with the *β*-glucosylasparagine linkage found in laminin [104]. This limitation may not exist for the archaeal AglB since there are instances in both *H. salinarum* and *H. volcanii* where the linking sugar is a hexose [7, 106].

**Figure 1:**
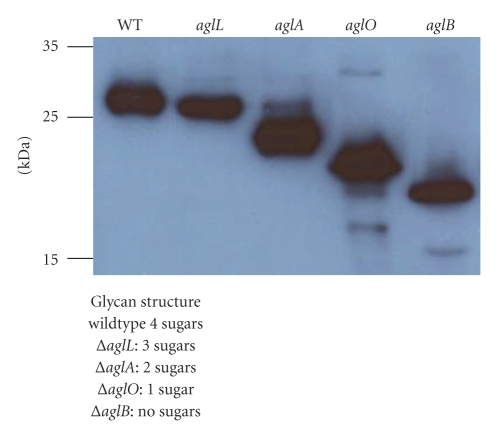
Immunoblot of wild-type *M. maripaludis* cells and mutants carrying deletions in various *agl* genes. Blots were developed with anti-flagellin antisera. Blot shows almost quantitative transfer of truncated glycans occurs.

 The archaeal AglB seems quite adept at transferring incomplete glycans, as mutants in the various glycosyltransferases needed to assemble the complete glycan nevertheless have proteins possessing the truncated glycan variants. In the case of flagellins of methanococci, there appears to be almost quantitative transfer of truncated glycans by AglB, as nonglycosylated flagellins are not observed in any abundance in western blots of glycosylation mutants (Figure 1). There are reports of PglB having relaxed specificity: certainly a variety of unnatural glycans including LPS O-antigens can be added and also incomplete glycans transferred [92, 109]. However, other reports in the native *Campylobacter* host indicate that many truncated glycans are not well transferred to the target proteins [97]. This relaxed specificity is in contrast to the oligosaccharyltransferase complex in yeast, where truncated glycans and nonnative structures are generally not acceptable substrates for transfer [108, 110, 111]. All oligosaccharyltransferases (the STT3 subunit of the eukaryotic OT complex, PglB and AglB) contain the conserved motif (WWDYG). Mutations in this conserved motif result in loss of N-linked glycosylation confirming its essential role in catalysis [89, 95].

The target for transfer of the assembled glycan is always an Asn residue in a conserved motif. This motif is N-X-S/T (X cannot be proline) in Eucarya but in *Campylobacter* this motif has an extended version, D/E-Z-N-X-S/T (Z and X cannot be proline) [112]. Examination of the sequences around the N-linked sequon in Archaea revealed that the bacterial extended motif is not found and indeed the simpler eukaryotic motif was used [113]. Interestingly, there is an unexplained variation found in the S layer of *H. salinarum, *where the position 2 Asn residue is occupied with a repeating unit N-linked glycan. All other sites in the protein where N-linked glycosylation occurs are occupied with an oligosaccharide glycan. Site-directed mutagenesis of that Asn-2 motif's serine (position 4) to valine, leucine, or asparagine did not prevent subsequent N-glycosylation at that site [114]. No suitable explanation for this interesting finding has been presented. Initial suggestions that perhaps there were multiple oligosaccharyltransferases in *H. salinarum* were not substantiated by subsequent genome sequencing, where only a single oligosaccharyltransferase gene was found [4]. How the cell differentiates between sites that receive the oligosaccharide glycan or the repeating unit glycan is unknown. 

The site of transfer of the glycan to the target proteins was shown to be external to the cell in *Halobacterium* [7]. Treatment of cells with bacitracin, which does not cross the cell membrane, inhibited the transfer of the repeating unit glycan but not the oligosaccharide glycan. Since bacitracin is known to inhibit glycosylation by binding to lipid pyrophosphates, this result suggested that assembly of the repeating unit glycan occurred using a lipid pyrophosphate carrier while the oligosaccharide glycan used a lipid monophosphate carrier. Transfer of glycan to a synthetic peptide that contained the N-linked target sequon, but was unable to cross the cytoplasmic membrane, was also shown. These data support the extracellular location of the AglB catalytic site. 

Knockouts of the oligosaccharyltransferase gene *aglB* in *M. maripaludis *[18, 19]*, M. voltae* [18], and *H. volcanii* [20] have demonstrated a marked reduction in target protein molecular weight consistent with a nonglycosylated target, as expected for cells lacking oligosaccharyltransferase activity. However, only with the oligosaccharyltransferase of *Pyrococcus furiosus* has an in vitro assay demonstrated that this subunit can catalyze the transfer of the glycan to the target sequon on peptides [17]. Work on the first crystal structure of any oligosaccharyltransferase revealed that the *P. furiosus* enzyme possessed a novel conserved motif (DK motif, Asp-X-X-Lys), which was subsequently confirmed in the yeast Stt3 subunit to be important for catalysis [17].

## 4. Glycan Structures

**Figure 2:**
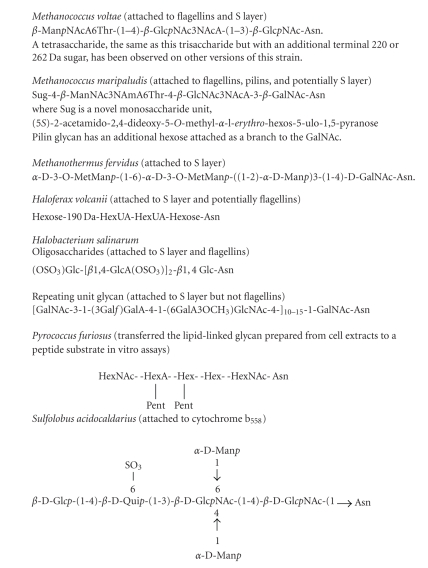
Structures of N-linked glycans in various Archaea.

N-linked glycan structures have been determined from a limited number of glycoproteins from a variety of archaea (Figure 2). However, only in the cases of *M. voltae, M. maripaludis, *and *H. volcanii* have genetic studies of the glycan assembly and biosynthesis accompanied the structural determination. 

### 4.1. *Methanococcus voltae*

Mass spectrometry, in combination with NMR analysis, was utilized to reveal the structure of the glycan N-linked to flagellins and the S layer protein in *M. voltae*. All the potential N-linked sequons (Table 2) of the four flagellin structural proteins (FlaA, FlaB1, FlaB2, and FlaB3) were occupied (1 site was not identified in the coverage of the protein) [9]. The glycan structure was revealed to be a novel N-linked trisaccharide of mass 779 Da with the structure *β*-Man*p*NAcA6Thr-(1–4)-*β*-Glc*p*NAc3NAcA-(1–3)-*β*-Glc*p*NAc-Asn [9]. The glycan is attached to asparagine via N-acetylglucosamine, with the second sugar being a diacetylated glucuronic acid and the terminal sugar being an unusually modified N-acetylmannuronic acid substituted with a threonine residue at position 6. (Sugar abbreviations: GlcNAc, 2-acetamido-2-deoxyglucose; GlcNAc3NAcA, 2,3-diacetamido-2,3-dideoxyglucuronic acid; ManNAcA, 2-acetamido-2-deoxymannuronic acid.)

Recent studies have shown that other versions of *M. voltae* strain PS can also glycosylate both the flagellin and S layer protein with a tetrasaccharide composed of the previously reported trisaccharide but with an additional residue of either 220 or 262 Da mass [64].

### 4.2. *Methanococcus maripaludis*

The glycan attached to flagellins in *M. maripaludis* was determined by mass spectrometry and NMR techniques to be a tetrasaccharide of mass of 1036 Da, related to the structure previously determined for the *M. voltae* glycan but with significant differences [13]. The structure was demonstrated to be Sug-4-*β*-ManNAc3NAmA6Thr-4-*β*-GlcNAc3NAcA-3-*β*-GalNAc-Asn (where Sug is a novel monosaccharide unit (5*S*)-2-acetamido-2,4-dideoxy-5-*O*-methyl-*α*-l-*erythro*-hexos-5-ulo-1,5-pyranose). An obvious difference in the two *Methanococcus* glycans is that *M. maripaludis* uses N-acetylgalactosamine (GalNAc) as the linking sugar while *M. voltae* employs N-acetylglucosamine. In addition, the third sugar was identified as a variant of the terminal (third) sugar in the *M. voltae* glycan by the addition of a 3-acetamidino group. Finally, the terminal sugar in the *M. maripaludis* glycan was particularly labile and, in addition, is unique in being the first example of a naturally occurring diglycoside of an aldulose [13]. The N26 site of processed flagellins FlaB1 and FlaB2 (potential glycosylation site 38 in the unprocessed flagellins with attached signal peptide; Table 2) which represent the first potential N-linked sequon in these *M. maripaludis *flagellins was not glycosylated [13]. This was unexpected since three of the *M. voltae* flagellins have an identically located N-glycosylation site, and in all cases, these are occupied by glycan [9] (Table 2).

### 4.3. *Haloferax volcanii*

In *Haloferax volcanii*, seven potential N-linked sequons are found within the S-layer glycoprotein (Table 2), and mass spectrometry provided insight into the composition of the glycan decorating at least two of these seven potential sites [12, 106]. The glycan was identified as a pentasaccharide comprised of two hexoses, two hexuronic acids, and an additional 190 Da saccharide attached via a hexose to Asn13 and Asn83 of the S layer protein [12]. The sequence of the pentasaccharide linked to Asn13 was determined to be hexose (Hex)-X-hexuronic acid (HexA)-HexA-Hex-peptide (Figure 2), where X is a 190 Da moiety recently revealed to be a methyl ester of hexuronic acid [115]. 

This structure is drastically different from the initial report of the N-linked glycan, attached at four of the potential seven sites, as a linear chain of 10 *β* 1,4-linked glucose residues with no amino sugars or uronic acids [11]. 

Analysis of the sequenced *H. volcanii* genome has also revealed the presence of two typical flagellin genes which have potential N-linked sites (Table 2).

### 4.4. Other Archaeal N-Linked Glycan Structures

The S layer protein of *Halobacterium salinarum* [3] is a truly remarkable protein with two different N-linked glycans (Figure 2), as well as a sulfated disaccharide of glucose and galactose attached in an O-linkage to threonine residues near the C-terminus of the molecule. One of the N-linked glycans is a repeating unit glycan attached via N-acetylgalactosamine to the very N-terminus of the protein at an asparagine at position 2. This pentasaccharide repeating unit (10–20 repeats) contains unusual features including a methylated galacturonic acid and a furanosidic galactose linked peripherally to a linear chain of GalNAc-GalA-GalNAc [116]. The second N-linked glycan, found in about 10 copies per protein, is a sulfated oligosaccharide linked to asparagine via glucose and this glycan is also found on the flagellins (Table 2). The glucose linking sugar is extended by 2-3 *β*-1,4 linked glucuronic acids, which are sometimes replaced with iduronic acid. 

An N-linked hexasaccharide glycan with the structure *α*-D-3-O-MetMan*p*-(1-6)-*α*-D-3-O-MetMan*p*-((1-2)-*α*-D-Man*p*)3-(1-4)-D-GalNAc-Asn (Figure 2) was reported on the S layer of the hyperthermophilic methanogen, *Methanothermus fervidus* [8]. As with the repeat unit glycan of *H. salinarum* and the glycan of *M. maripaludis*, the linking sugar is N-acetylgalactosamine. The protein itself has 20 potential N-linked sequon sites with half of them occupied with the glycan. 

Cytochrome b_558–566_ in *Sulfolobus acidocaldarius* [15] is a highly glycosylated membrane protein modified at least 7 positions with a hexasaccharide. The glycan consists of 2 residues each of mannose and N-acetylglucosamine and one each of glucose and the rare sugar, 6-deoxy-6-sulfoglucose (6-sulfoquinovose) (Figure 2). The linkage is through N-acetylglucosamine. A cytoplasmic membrane protein in *Thermoplasma acidophilum* that contains a highly branched glycan, composed mainly of mannose, N-linked through an N-acetylglucosamine has also been reported [16]. In addition, it was shown in *Pyrococcus furiosus* that the purified oligosaccharyltransferase could transfer a lipid-linked heptasaccharide prepared from *P. furiosus* cells to an Asn within a sequon Asn-X-Thr/Ser contained in a peptide substrate [17]. The heptasaccharide is branched and consisted of two N-acetylhexosamines, two hexoses, a hexA, and two pentoses. 

The cell sheath of *Methanosaeta concilii, *the outermost layer of the complex cell envelope of this organism, was reported to be a glycoprotein with the glycan N-linked via a rhamnose residue [117]. However, the rhamnosyl peptide identified was reported as Rha-Asn-Glu-Gly-Ser and not the canonical Asn-X-Ser/Thr sequon.

## 5. Assembly of the Glycans

### 5.1. *Methanococcus voltae*

Most of the enzymes needed to assemble the glycan N-linked to flagellins and S-layer have been identified in both *M. voltae* and *M. maripaludis*. The glycan of *M. voltae* cultured long term in the Jarrell lab has a three sugar component [9], although a fourth sugar has been detected in other versions of this strain [64]. Likely the Jarrell version of the strain developed a spontaneous mutation affecting the biosynthesis or addition of the fourth sugar. Utilizing a very primitive genetic system that allowed only insertional inactivation of targeted genes, Chaban et al., in a series of papers, identified the glycosyltransferases and the oligosaccharyltransferase needed for its assembly. The initial study inactivated a gene designated *aglA* which resulted in faster migrating flagellins and S layer proteins than observed in the wild type [18]. When flagella from the *aglA* mutant were isolated and subjected to mass spectroscopy analysis, it was determined that the terminal sugar, the modified mannuronic acid, was missing, indicating a role for AglA in the transfer of this last sugar onto the glycan. The linking sugar for the *M. voltae* glycan is N-acetylglucosamine, and a gene thought to catalyze the addition of this first sugar onto the dolichol lipid carrier was identified in the *M. voltae* genome. Repeated attempts to inactivate this gene failed, indicating perhaps its essential nature in *M. voltae*. This gene, designated *aglH*, was the only one in the *M. voltae *genome that belonged to Pfam family PF00953 (glycosyltransferase family 4), the family that includes the eukaryotic gene *alg7,* encoding the enzyme N-acetylglucosamine-1-phosphate transferase that attaches N-acetylglucosamine to the eukaryotic dolichol lipid carrier, in the first dedicated step of the N-glycosylation process. Evidence that the *M. voltae* gene did, in fact, encode the first glycosyltransferase for assembly of the archaeal glycan was obtained when it was shown that *aglH* could complement a conditionally lethal mutation in *alg7* in *Saccharomyces cerevisiae* [118]. With AglA attaching the last of the three sugars and AglH attaching the first, this left identification of the glycosyltransferase for the second sugar. Unexpectedly, two genes annotated as glycosyltransferases were shown to be involved in this step of glycan assembly [64]. Inactivation of either *aglC* or its adjacent gene *algK* resulted in flagellins that had molecular masses smaller than those of *aglA* mutants missing the third sugar but higher than flagellins from that of an oligosaccharyltransferase mutant that would be completely nonglycosylated. Both AglC and AglK are involved in either transfer or biosynthesis of the second sugar of the glycan. 

A key enzyme in the N-glycosylation process is the oligosaccharyltransferase which transfers the assembled glycan to the target protein. This enzyme has a highly conserved catalytic motif (WWDXG) which simplifies its identification. In *M. voltae,* this gene, designated *aglB*, was disrupted, and the mutants carrying this disruption has S layer protein and flagellin molecular masses smaller than those found in any of the other *agl* mutants, consistent with this enzyme being responsible for the transfer of the N-glycan [18]. 

Since the oligosaccharyltransferase gene could be inactivated, N-linked glycosylation appears to be a nonessential pathway in *M. voltae*. This means that the inability to inactivate the first glycosyltransferase is unrelated to its role in N-linked glycosylation and must lie in its role in another pathway essential for the cell, unless inactivation of this gene at this site of the chromosome is simply highly unfavoured. One such essential pathway may be in the biosynthesis of the methanogenesis cofactor, component B (7-mercaptoheptanoylthreonine) [119] which has been reported to contain an acetamido sugar disaccharide consisting partly of N-acetylglucosamine [120]. This cofactor is essential for the terminal step in methanogenesis catalyzed by methyl CoM reductase [120], and its defective biosynthesis would be lethal. Interestingly, it was not possible to delete the corresponding gene in *M. maripaludis* either [19], even though the linking sugar in *M. maripaludis* is not N-acetylglucosamine but N-acetylgalactosamine [13]. This further suggests that the essential role that enzyme plays lies outside the N-linked glycosylation system. 

No flippase, proposed to be necessary to flip the assembled lipid-linked glycan to the external face of the cytoplasmic membrane before its attachment to the target protein by the oligosaccharyltransferase, was identified in the *M. voltae *system. Candidate genes were either inactivated without the predicted phenotype or were unable to be inactivated [18, 19]. 

**Figure 3:**
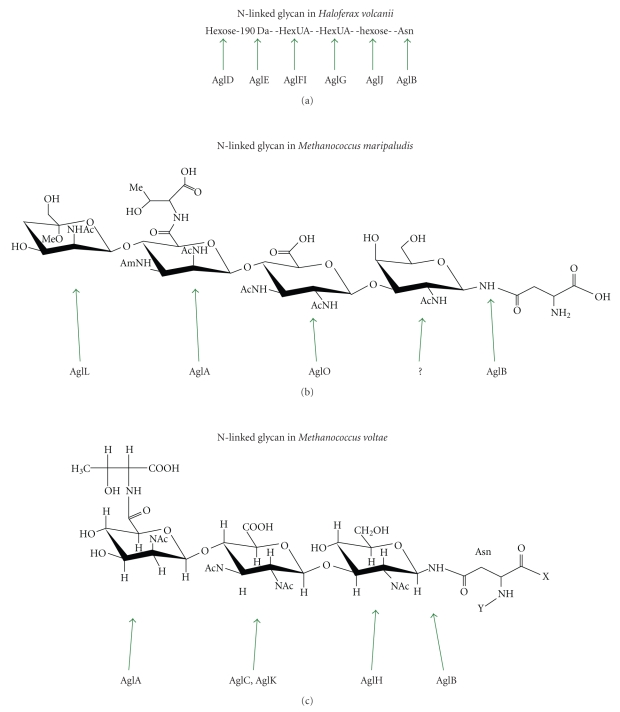
Stage of action of various glycosyltransferases and oligosaccharyltransferase in the N-linked glycan assembly of *Haloferax volcanii*, *Methanococcus maripaludis,* and *Methanococcus voltae*.

A summary of the site of action of the *M. voltae* glycosyltransferases and oligosaccharyltransferase is shown in Figure 3.

### 5.2. *M. maripaludis*

 In 2005, a genetic system was reported for an *M. voltae* relative, that is, *M. maripaludis*. This system allowed for a markerless inframe deletion of targeted genes and their subsequent complementation by plasmid borne versions [121]. As a result, this organism became a natural choice for continued investigations on the N-linked glycosylation system in archaea. Structural work on the glycan of *M. maripaludis *flagellins revealed interesting differences, including its tetrasaccharide nature, its unique terminal sugar, and the fact that, unlike the *M. voltae* glycan, its attachment to the protein was via N-acetylgalactosamine [13]. Attempts to identify the likely 4 glycosyltransferases involved in its assembly began with targeting the approximately dozen annotated glycosyltransferase genes in the published genome. One locus was found to harbour three glycosyltransferases involved in assembly of the N-linked glycan of *M. maripaludis*. These genes, MMP1079, MMP1080 and MMP1088 designated *aglO*, *aglA,* and *aglL,* respectively, were separated by other genes, some of which were later shown to be involved in the biosynthesis of the component sugars (see below). Located elsewhere on the genome was the oligosaccharide transferase, *aglB* (MMP1424). Deletions of each of these genes lead to decreases in the molecular mass of the flagellins compared to wild-type cells, with the *aglB* mutant, presumably completely lacking the N-linked glycan, possessing flagellins with the lowest mass [19]. In order, the flagellin masses decreased in size from wild-type to the *aglL*, *aglA*, *algO,* and *aglB *mutants. Mass spectrometry analysis of purified flagellins from the *aglL* mutant indicated it lacked the terminal sugar as well as the threonine attached to the third sugar. Similar analysis of flagella from the *aglA* mutant showed only the first two sugars present on the glycan. MMP1080 was designated *aglA* since it is 55% similar to the *M. voltae aglA* sequence [18], and the two enzymes transfer a very similar modified mannuronic acid residue to an identical diacetylated glucuronic acid (second sugar). All of the deleted genes could be complemented with plasmid borne copies resulting in wild-type glycan formation. The MS data on the structure of the *aglL* mutant glycan is unusual in that two significant changes were noted. The fourth sugar was entirely missing but also the threonine attachment to the third sugar was missing. In theory, AglL could be responsible for either of these two changes (or both). Because of the strong similarity of AglL to known glycosyltransferases, we believe that it is transferring the final sugar and not the threonine. This would suggest that the threonine attachment likely occurs to the third sugar only after the four-sugar glycan has been completed. Without the attachment of the fourth sugar in the *aglL* mutant, the threonine cannot be attached to the third sugar. The enzyme responsible for the threonine attachment has not been identified. 

These studies identified the oligosaccharyltransferase and three of the four needed glycosyltransferases. Still unaccounted for is the glycosyltransferase responsible for attaching the first sugar to the dolichol lipid carrier. In* M. voltae,* this was identified as a eukaryotic *alg7* homologue. However, in *M. maripaludis* the linking sugar is not N-acetylglucosamine but rather N-acetylgalactosamine. The glycosyltransferase transferring an N-acetylgalactosamine to the dolichol lipid to start assembly of the glycan has not been identified. Recently, an epimerase that converts N-acetylglucosamine-P-P-undecaprenol to N-acetylgalactosamine-P-P-undecaprenol in O-antigen biosynthesis in *E. coli* was reported [122]. While such an activity has not been reported for converting N-acetylglucosamine-P-P-dolichol to N-acetylgalactosamine P-P-dolichol in eukaryotes, the possibility exists that AglH assembles an N-acetylglucosamine-dolichol precursor that is subject to epimerization to create the final N-acetylgalactosamine version. A BLAST search using the *E. coli *epimerase did identify a strong hit in *M. maripaludis,* MMP1090, which when deleted resulted in a large downshift in flagellin molecular weight, suggesting a flagellin with a much reduced glycan chain (unpublished data). All of the other annotated glycosyltransferase genes of *M. maripaludis* have been targeted for deletion in efforts to identify this first glycosyltransferase. However, all potential candidates successfully deleted did not yield the expected phenotype (flagellin molecular weight identical to that of *aglB* mutants). Several candidate genes were not successfully deleted, however, and additional glycosyltransferase candidates have been identified manually in the complete sequence (unpublished data). *M. voltae, M. maripaludis* and *H. volcanii* encode many more glycosyltransferases than are needed solely for N-linked glycan assembly. These are presumably involved in other processes, such as O-linked glycosylation and perhaps glycolipid and cofactor synthesis since, at least in methanogens, sugars can be shared among those processes [120, 123]. 

As with *M. voltae*, attempts to identify the putative flippase in *M. maripaludis* have been unsuccessful, either due to an inability to delete targeted candidates or because targeted candidate genes for the flippase that were deleted did not have the expected phenotype. 

A summary of the site of action of the *M. maripaludis* glycosyltransferases and oligosaccharyltransferase is shown in Figure 3.

### 5.3. *Haloferax volcanii*

The Eichler lab has presented a series of papers that identify a role for many *agl* genes in the biosynthesis, assembly, and transfer of a pentasaccharide glycan N-linked to the S layer protein at positions Asn 13 and Asn 83 [12]. The study of the glycosylation of this target is complicated by the presence of O-linked glycans as well. The glycan attached to S layer in *H. volcanii *consists of a hexose, followed by 2 hexuronic acids, a 190 Da sugar (a methyl ester of a hexuronic acid), and finally another hexose with the link to the Asn being via a hexose [12], an unusual N-linkage first reported in *Halobacterium salinarum* [93]. 

Unlike in *Methanococcus* species where the *aglB* oligosaccharyltransferase is located at a distance from many of the glycosyltransferases needed to assemble the glycan, in *H. volcanii* one large *agl* cluster spanning nearly 20 kb includes all but one of the genes (*aglD*) identified as being involved in the N-glycosylation process [124, 125]. A number of gene products have been assigned glycosyltransferase function: AglG for addition of the hexuronic acid at position 2, AglI for addition of the hexuronic acid at position 3 [126], AglE for the addition of the 190 Da sugar [127], and AglD for the addition of the terminal hexose [12]. In addition, there is evidence for AglJ being the glycosyltransferase responsible for transfer of the first sugar. 

The topology of certain enzymes, namely, AglJ and AglD, involved in early and late stages of the N-glycosylation process was demonstrated to be with their N-terminal domains (likely containing their catalytic sites) facing the cytoplasm, indicating that assembly of the glycan on the dolichol lipid carrier occurs on the cytoplasmic face of the cytoplasmic membrane before flipping to the external face for transfer onto the target proteins via AglB [100]. 

A summary of the site of action of the *H. volcanii* glycosyltransferases and oligosaccharyltransferase is shown in Figure 3.

Genetic analysis of the N-linked system in other archaeal species has not yet been reported, even for those in which N-linked glycan structures have been determined. This is due, to some cases, to the complete lack of a suitable genetic system.

## 6. Biosynthesis Genes

Although a biosynthetic pathway for sugar residues involved in the glycan structure displayed in bacteria is well characterized [2], it is only very recently that the enzymes responsible for the process in Archaea have been discovered. Currently, the majority of data regarding the glycan biosynthesis pathway is focused around two archaeal species, *M. maripaludis* and *H. volcanii.*

### 6.1. *M. maripaludis*

Recent *in vitro* experimentation, using heterologously expressed and purified proteins predicted to be involved in glycosylation, helped to describe the biosynthesis of the acetamido sugar subunit precursors in *M. maripaludis* [119]. MMP1680 was shown to transamidate and epimerize the reaction of fructose-6-phosphate and L-glutamine to glucosamine-6-phosphate and glutamate, respectively. Additionally, the conversion of glucosamine-6-phosphate to glucosamine-1-phosphate through the activity of MMP1077 was demonstrated. The immediate neighbor of MMP1077, MMP1076 (GlcN-1P uridyltransferase/acetylase) acts in a bifunctional manner, both acetylating the 2-amino group and facilitating the transfer to UTP producing UDP-*N*-acetylglucosamine. This sugar subunit is the immediate precursor for several different biosynthetic pathways resulting in the sugar residues that are added to the glycan structure. Interestingly, *N*-acetylglucosamine is the linking residue of the N-glycan in *M. voltae* [9] while N-acetylgalactosamine, which could be derived from N-acetylglucosamine through the activity of an epimerase, fulfills this role in* M. maripaludis *[13], thus emphasizing the importance of the description of this pathway to understanding glycosylation in Archaea. MMP0705 (WecB) catalyses the isomerization of UDP-*N*-acetylglucosamine to UDP-N-acetylmannosamine [119], and MMP0706, the UDP-ManNAc 6-dehydrogenase (WecC), oxidizes UDP- N-acetylmannosamine to UDP- N-acetylmannosaminuronate, a direct precursor to the third sugar of the *M. maripaludis* glycan. Additionally, component B has been reported to have an attached N-acetylmannosaminuronate *β*1-4 UDP-*N*-acetylglucosamine disaccharide, and, since component B is essential for methanogenesis, disruption of its sugar moiety may lead to an inactive form and cell death. Attempts to delete either MMP0705 or MMP0706 have so far been unsuccessful (unpublished data), indicating a possible essential role of these enzymes in the cell, likely in component B biosynthesis. 2-acetamido sugars such as those discussed have also been found in *M. voltae* glycolipids [123], while glycolipid structures in *M. maripaludis* have not been reported.

In addition to this *in vitro* work, techniques utilizing in-frame deletions of putative glycosylation genes have proven to be particularly useful. The first major finding reported in the biosynthetic pathway using this technique was the gene MMP0350. The gene, which shows strong sequence similarity to known acetyltransferases, appears to attach an acetyl group to the second sugar of the glycan, a diacetylated glucuronic acid. Lack of this modification resulted in an inability of the cells to attach the second sugar and left a truncated glycan of only 1 sugar [128]. 

An inframe deletion of MMP1081 resulted in a truncated glycan which was shown by mass spectrometry to be missing the acetamidino group of the third sugar as well as the entire fourth sugar (unpublished results). The homology to WpbG suggested MMP1081 as an acetamidino-transferase responsible for addition of this modification to the third sugar (unpublished results). The glycan of *M. voltae* strain PS has this sugar without the acetamidino group added. Unexpectedly, a strong homologue of MMP1081 is found in the genome of *M. voltae *A3. If this gene is also present and expressed in *M. voltae* strain PS, the strain in which the glycan structures have been determined, why the acetamidino group would not be added to the third sugar is unknown.

A second gene in this region has been experimentally confirmed to have a role in the glycosylation pathway. MMP1085, annotated as a methyl transferase, was shown to be responsible for the addition of a methyl group to the terminal sugar. Mutants in this gene were first detected to have a small downward shift in flagellin molecular weight in western blots, and subsequent mass spectrometry analysis of purified flagella from the MMP1085 mutant determined the lack of the methyl group (unpublished results). Several other genes in this locus are cotranscribed with either MMP1081 or MMP1085, and deletions in MMP1083 but not MMP1082 or MMP1087 appear to result in smaller flagellins in western blots suggesting that they also have defects in their glycan although this needs confirmation by mass spectrometry (unpublished data). 

A summary of the deletion mutants studied in *M. maripaludis* so far and their effects on glycan structure and the flagella system are summarized in Table 3.

### 6.2. *H. volcanii*

Several genes have been identified in the glycan biosynthesis pathway of the pentasaccharide expressed on the *H. volcanii* surface layer, through deletion analysis as well as *in vitro* biochemical characterization [115, 125]. The coordinated actions of AglM and AglF have been demonstrated. AglM functions as a UDP-glucose dehydrogenase, which catalyzes the generation of UDP-glucuronic acid from UDP-glucose, and is involved in the biosynthesis of the hexuronic acid at position 2 and likely also the one at position 3. AglF, a glucose-1-phosphate uridyltransferase, acts in conjunction with AglM in the biosynthesis of the hexuronic acid at position 3 but not, however, the one found at position 2 [125].

In addition, several other genes have been assigned roles in the N-linked glycosylation process, specifically *aglP*, *aglQ,* and *aglR,* based partly on their cotranscription with known *agl* genes [124]. Recently, Magidovich et al. [115] have confirmed the action of AlgP as an S-adenosyl-L-methionine-dependent methyltransferase as had been previously predicted through bioinformatics analysis [124]. This enzyme is responsible for the addition of a methyl group to the hexuronic acid found at position four of the *H. volcanii *glycan structure. A tagged version of AglP located to the cytoplasm and biochemical assays confirmed its ability to transfer methyl groups to membrane fragments of *H. volcanii* wild-type cells but not to those of an *aglE* mutant which lacks the 4th sugar. Interestingly, *aglP* mutants had only a tetrasaccharide attached to their S layer; apparently the methyl group addition to sugar 4 is required for subsequent attachment of the terminal sugar. Protein sequence comparisons between AlgP of *H. volcanii* and the previously discussed *M. maripaludis* methyltransferase MMP1085 suggest low sequence identity, likely due to structural differences of the modified sugar in each glycan.

These in vitro studies, along with those on the *P. furiosus* AglB [17, 129], represent significant steps forward in the analysis of the archaeal N-glycosylation mechanism.

A summary of the deletion mutants studied in *H. volcanii* so far and their effects on glycan structure, S layer, and cell growth are summarized in Table 4.

## 7. Effects of Glycan Modifications on Surface Structures

### 7.1. Flagella

Archaeal flagellin subunits have proven to be a rich resource as reporter proteins in the study of N-linked glycosylation in Archaea. Effects of deletions of genes involved in the N-glycosylation pathway of both *M. voltae *and *M. maripaludis *can typically be visualized by faster migration of flagellin subunits through SDS-PAGE and western blot analysis, due to a decrease in the molecular weight corresponding to the truncated glycan [18, 19]. Due to the relatively small size of flagellin subunits and the multiple sites of attachment of glycan, even very minor changes in the structure of the glycan have been resolved. 

It has been previously demonstrated that the flagella structure is dependent on having at least a two-sugar glycan attached to flagellin subunits in both *M. voltae *and *M. maripaludis* [18, 19]. Thus, mutants in *aglA,* the glycosyltransferase adding the third (terminal) sugar in the *M. voltae* glycan, are still flagellated while deletions in *algC* or *aglK* which are needed to add the second sugar, as well as in the oligosaccharide transferase, *aglB,* are completely nonflagellated [18, 64]. Similarly, for *M. maripaludis, *mutants that result in the loss of the terminal sugar (*aglL*) or the third sugar (*aglA*) are still flagellated while a mutant in *aglO*, responsible for transferring the second sugar or in *aglB,* is nonflagellated [19]. An acetyltransferase gene, MMP0350, likely responsible for addition of an acetyl group to the second sugar (the diacetylated glucuronic acid) [19, 128], results in flagellin with apparently a single sugar added, judged solely by apparent molecular weight comparisons to the corresponding molecular weights of various glycosyltransferase mutants. In accordance with the 2-sugar glycan rule, these mutants were also nonflagellated. 

Additionally, one major advantage of examining glycan modification on the flagella is the ability to perform motility assays, thus demonstrating the *in vivo* importance of glycan for the proper functioning of this particular surface structure. For cells that assemble flagella, this technique yields measurable differences in the swimming capabilities of strains harbouring disruptions in the glycosylation pathway when compared to wild-type cells. It has been previously reported that cell motility decreases in accordance with the degree of truncation from the wild-type glycan. *M. maripaludis* cells lacking the terminal sugar due to the deletion of *aglL* swim less distance than wild type in motility agar (0.25% agar), but a greater distance than cells which are lacking the third sugar due to inactivation of *aglA* [19]. In agreement with this, a mutant in MMP1081, demonstrated to be responsible for addition of an acetamidino group to the third sugar, swims a distance intermediate to that of the *aglA* and *aglL* mutants (unpublished results). 

Undermodified flagellins of *H. salinarum* were also found to result in aberrant effects on flagella stability. In this instance, a mutant that overproduced flagella and released them into the media was found to be producing flagellins of lower molecular weight compared to wild-type cells suggestive of an underglycosylation. The exact difference in glycan structure was not reported [14]. In addition, when bacitracin, a known inhibitor of glycosylation, was added to growth medium of *Methanococcus deltae*, the cells became nonflagellated with flagellin molecular weight shifted to a lower molecular weight, again suggestive of underglycosylation. No structures for the flagellin glycans of the wild-type or bacitracin-treated cells were determined [130].

### 7.2. Pili

In addition to the nonflagellated phenotype observed in the *M. maripaludis* acetyltransferase mutant MMP0350, an interesting result was elucidated from this study in relation to the pili in this species [128]. While the cells were typically lacking pili, analysis of the culture supernatants revealed the presence of apparently fully formed pilus structures, suggesting that the glycan defect had not interfered with pili formation but yet had prevented proper attachment of the pili to the cell surface. While this study was interesting in being the first reported case of a gene deletion that resulted in an effect on pili in any archaeal species, it also suggested a more diverse role for the glycan in the surface structures of archaea than previously assumed.

### 7.3. S Layer

S layer proteins are the other archaeal targets that have proven to be useful reporter proteins for the study of the *in vivo *function of the N-linked glycan. Mutants producing various truncated glycans have been shown to have altered migration of their corresponding S-layer proteins through SDS-PAGE in both *M. voltae* [18] and *H. volcanii* [125, 126]. While in *M. voltae* all defects in glycan structure resulted in altered S layer migration in SDS-PAGE, in the case of *H. volcanii*, certain deletions of genes confirmed to be involved in the glycosylation pathway did not demonstrate the smaller molecular weight migration that other gene deletions did [20, 127]. This is somewhat puzzling since only 2 potential sites (at least one is occupied) are found for N-linked glycosylation in *M. voltae *while 7 (at least 2 of which are used) are found in the S layer protein of *H. volcanii.* In *M. maripaludis,* where there is only a single potential N-glycosylation site, no difference in the S layer apparent molecular weight was noticed in glycosylation defective mutants compared to the wild-type, even in an *aglB* mutant. However, it is not known whether the N-linked sequon actually is occupied in the *M. maripaludis* S layer protein. 

In both *Methanococcus* and *Haloferax,* the S layer is only component of the cell envelope external to the cytoplasmic membrane. Due to the key role of the S layer in these archaea, the specific contribution of glycosylation to the stability and function of the S layer has been addressed extensively in the case of *H. volcanii* and its various *agl* mutants. 

S-layer proteins in *H. volcanii *strains lacking AglD, the glycosyltransferase responsible for the addition of the terminal sugar, were found to have a defect in the symmetry and the proper assembly of the S layer as compared to wild-type cells [12]. In addition, both AglD mutants and those deficient in AglB, the oligosaccharide transferase, were observed to have substantially deficient growth in media containing higher concentrations of salt compared to those expressing the normal glycan, suggesting a physiological role for the modifications in this halophile. However, analysis of mutants carrying a deletion of a second glycosyltransferase *aglE*, which is responsible for addition of the second sugar residue, showed no decrease in growth rates at similar salt concentrations [127]. Similar results in which growth rates were unaffected were later reported in mutants carrying deletions in *aglF*, *aglG*, and *aglI* [126]. Another measure of S layer stability tested was the susceptibility of the S layer protein of various *agl* mutants to proteases. Interestingly, S layers of cells deleted for *aglD* were more susceptible to protease treatment than cells deleted for *aglB* and thus missing the entire glycan. Mutants carrying deletions of *aglM*, responsible for addition of the second sugar, had both reduced S layer molecular weight and increased susceptibility to proteases [125]. It has been suggested that N-glycosylation may be an adaptive response in *H. volcanii* since transcription of various *agl* genes differs under different growth conditions [125].

## 8. Model of Assembly of Archaeal Glycosylated Flagellins into Flagella Filaments

The N-linked glycosylation system is intimately involved in flagellation in *Methanococcus*. N-linked glycosylation is one of two major posttranslational modifications that flagellins undergo in *M. voltae and M. maripaludis*, the other being signal peptide removal. Like bacterial type IV pilins [45] which form a pilus involved in surface movement called twitching [131], archaeal flagellins are synthesized as preproteins with class III signal peptides [132] that are removed by a dedicated signal peptidase in both systems: PilD in type IV pilus systems [133] and the homologous FlaK/PibD in archaeal flagella systems [46, 47, 50]. Unlike bacterial flagella, the archaeal flagella are not hollow, and there is not a space large enough to accommodate the passage of new subunits to the distal end [43, 44]. Thus, as was suggested by Jarrell et al. [32], new subunits appear to be incorporated at the base, like pilins. 

Both N-linked glycosylation and the signal peptide removal can occur independently of each other. Flagellins are transported to the cytoplasmic membrane, likely guided by chaperones [134], and then undergo glycosylation and processing by the preflagellin peptidase. The posttranslationally modified flagellins are then incorporated at the base of the structure, likely through the activity of FlaI, a homologue of the type IV pilin assembly ATPase PilT [48]. The platform for assembly may be FlaJ, a homologue of the conserved membrane protein PilC in the type IV pilin system [49]. 

**Figure 4:**
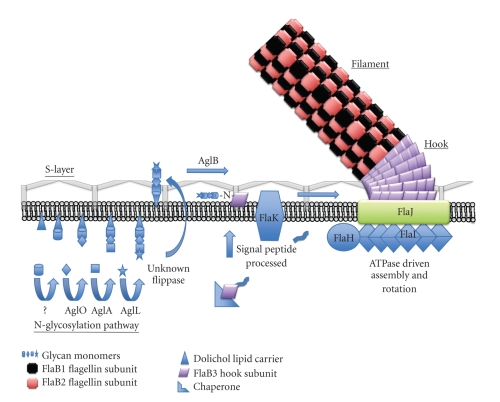
Model of assembly of *M. maripaludis* flagella, incorporating glycan synthesis and signal peptide removal.

A hypothetic model of how the N-linked glycosylation system and assembly of flagellins into flagella are linked is depicted in Figure 4.

Since the S layer protein of *M. voltae* has been shown to be decorated with the same glycan as flagellins and both flagellins and S layers of *H. salinarum* are decorated with the same N-linked oligosaccharide, it has been assumed that the N-linked system is shared by all glycosylated proteins within a given cell. While this is likely true, there is data already presented in *H. salinarum* that this would be an oversimplification. As pointed out, the S layer protein in this organism has two different N-linked glycans attached at different sequon locations, and only the oligosaccharide glycan is found on flagellins of *H. salinarum* [7], indicating some selectivity in transfer of the glycan even though the transfer is likely to be by a single oligosaccharyltransferase. A novel feature of isolated pili of *M. maripaludis* is that the structural proteins contain a glycan identical to that of the flagellins but with an additional branched hexose attached to the N-acetylgalactosamine (unpublished results).

## 9. Conclusions

N-linked glycosylation in archaea has been demonstrated to occur on a number of proteins, especially those of surface proteins comprising S layers, flagellins, and pilins. Interference with the N-glycosylation system can have severe effects on flagella assembly and function as well as S layer stability. Study of N-glycosylation in archaea has shown a wide variety of sugar protein linkages as well as the presence of novel sugar components. These findings have indicated that the archaea are a vast untapped reservoir of biosynthetic and assembly genes for construction of tailored glycoproteins. Even more so than the potential of bacterial glycosylation systems [135], archaeal diversity may have important applications to nanobiotechnology. Since archaeal glycosylation may aid in the stability of proteins in extreme environments [15, 136], useful biotechnology applications of these modifications to enhance stability of nonarchaeal proteins under extreme conditions may be possible. It has already been demonstrated that an archaeal glycosyltransferase, that is, AglH, can function effectively in yeast cells [118]. 

In addition, it has been clearly demonstrated already that study of the archaeal version of this important posttranslational modification can lead to substantial progress in understanding aspects of the eukaryotic version. A case in point is the discovery of an important new motif in the oligosaccharyltransferase of *Pyrococcus*; subsequent mutational study of the motif in yeast Stt3 revealed its essential role in catalysis [17]. 

Clearly study of N-glycosylation in Archaea promises to yield exciting findings not only in archaeal physiology but also in eukaryotic biology and biotechnology.

## Figures and Tables

**Table 1:** Comparison of N-glycosylation in the three domains.

Trait	Eukarya	Bacteria	Archaea
Linking sugar	GlcNAc	Di-acetyl-bacillosamine	GlcNAc, GalNAc, Glucose
Lipid carrier	Dolichol-PP	Undecaprenol-PP	Dolichol-PP, Dolichol-P
Flippase	Rft1; ATP-independent (Role of Rft questioned)	PglK ATP-dependent	Unknown
Sequon in target protein	N-X-S/T (X not P)	D/E-Z-N-X-S/T (Z, X not P)	N-X-S/T (X not P), N-X-N/L/V (X not P)
Oligosaccharyl- transferase	STT3 subunit of multimeric complex	PglB single subunit (Stt3 homologue)	AglB single subunit (Stt3 homologue)
Oligosaccharyl- transferase catalytic type	E-type	B-type	A, B, and E-type
Site of assembly of glycan	Cytosolic face of ER membrane	Cytoplasmic face of cytoplasmic membrane	Cytoplasmic face of cytoplasmic membrane
Site of glycan transfer	Lumenal face of ER membrane	External face of cytoplasmic membrane	External face of cytoplasmic membrane

**Table 2:** Potential sites for N-glycosylation in selected archaeal proteins.

Protein	Length in amino acids (including signal peptide)	Demonstrated glycosylation	Potential glycosylation sites (amino acid position)
*M. voltae*			
FlaA	219	Yes	38, 175
FlaB1	218	Yes	38, 71, 77, 115, 136
FlaB2	216	Yes	38, 72, 77, 113, 172, 208
FlaB3	239	Yes	115, 128
S layer	581	Yes	107, 137

*M. maripaludis*			
FlaB1	211	Yes	38, 12, 128, 168
FlaB2	216	Yes	38, 78, 122, 131, 136
FlaB3	210	Yes	150, 175
MMP1685 Pilin	74	Yes	39, 49, 52, 71
MMP0233 Pilin	129	No	63, 87, 94, 108
MMP0236 Pilin	132	No	34, 74, 83, 94, 98, 119, 126
MMP0237 Pilin	151	No	72, 82, 89, 95
S layer	575	No	535

*H. volcanii*			
FlaA1	213	No	70, 115, 128, 134, 162, 172
FlaA	220	No	78, 95, 112, 124
S layer	827	Yes	47, 114, 308, 313, 404, 532, 766

*H. salinarium*			
FlgA1	196	Yes	84, 97, 125
FlgA2	194	Yes	80, 93, 126
FlgB1	193	Yes	80, 93, 123
FlgB2	196	Yes	84, 97, 125
FlgB3	193	Yes	80, 93, 123
S layer	852	Yes	36, 51, 327, 339, 398, 438, 513, 643, 727, 751, 787, 811, 815

**Table 3:** Effects of *agl* gene deletions on flagellin glycosylation in *M. maripaludis. *

Gene deletion	Function	Glycan structure	MW shift of flagellin	Flagella	Effect on motility
*aglL*	4th GTase	Missing 4th sugar and Thr on 3rd sugar	Yes	Yes	Decreased compared to WT
*aglA*	3rd GTase	Missing 3rd and 4th sugars	Yes	Yes	Decreased compared to aglL mutant
*aglO*	2nd GTase	Missing 2nd, 3rd, and 4th sugars	Yes	No	Nonmotile
*aglB*	OTase	No glycan	Yes	No	Nonmotile
*MMP0350*	Acetyl transferase	Missing 2nd, 3rd, and 4th sugars	Yes	No	Nonmotile
*MMP1081*	Acetamidino transfer	Missing 4th sugar and acetamidino group of 3rd sugar	Yes	Yes	Decreased compared to WT
*MMP1085*	Methyl transferase	Missing methyl group of 4th sugar	Yes	Yes	To be determined

**Table 4:** Effects of *agl* gene deletions on S layer glycosylation in *H. volcanii. *

Gene deletion	Function	Glycan structure	Growth in high salt	MW shift of S layer	Susceptibility of S layer to proteases	Shedding of S layer	S layer symmetry
*aglB*	OTase	No glycan	Deficient	Yes	Same as WT	Increased	No effect
*aglD*	5th GTase	Missing 5th sugar	Deficient	Yes	Less than WT	Decreased	Symmetry defect
*aglE*	4th GTase	Missing 4th and 5th sugars	No effect	No	Same as WT	Same as WT	No effect
*aglF*	Glucose-1-P Uridyltransferase (sugar 3)	Mix of mono and disaccharides	No effect	Yes	More than WT	Same as WT	No effect
*aglG*	2nd GTase	Missing last 4 sugars	No effect	Yes	More than WT	Same as WT	No effect
*aglI*	3rd GTase	Mix of mono and disaccharides	No effect	Yes	More than WT	Same as WT	No effect
*aglM*	UDP-Glucose dehydrogenase (sugars 2 and 3)	Missing last 4 sugars	Not reported	Yes	More than WT	Not reported	Not reported
*aglP*	Methyl transferase (sugar 4)	Missing 5th sugar and methyl group of sugar 4	Not reported	Not reported	More than WT	Not reported	Not reported
